# Acceleration of an aldo-keto reductase by minimal loop engineering

**DOI:** 10.1093/protein/gzu021

**Published:** 2014-07

**Authors:** C. Krump, M. Vogl, L. Brecker, B. Nidetzky, R. Kratzer

**Affiliations:** 1 Institute of Biotechnology and Biochemical Engineering, Graz University of Technology, Member of NAWI Graz, Petersgasse 12, 8010 Graz, Austria; 2 Institute of Organic Chemistry, University of Vienna, Währingerstraße 38, 1090 Vienna, Austria; 3 Austrian Centre of Industrial Biotechnology, Petersgasse 14, 8010 Graz, Austria

**Keywords:** Aldo-keto reductase, chiral ethyl mandelates, enzyme engineering, saturation transfer difference NMR

## Abstract

Aldo-keto reductases tighten coenzyme binding by forming a hydrogen bond across the pyrophosphate group of NAD(P)(H). Mutation of the hydrogen bonding anchor Lys24 in *Candida tenuis* xylose reductase prevents fastening of the “safety belt” around NAD(H). The loosened NAD(H) binding leads to increased turnover numbers (*k*_cat_) for reductions of bulky-bulky ketones at constant substrate and coenzyme affinities (i.e. *K*_m Ketone_, *K*_m NADH_).

Aldo-keto reductases (AKRs) constitute a protein superfamily of mainly NAD(P)(H)-dependent oxidoreductases. General features of these enzymes are broad substrate specificity and high stereoselectivities (Kaluzna *et al.*, 2004), thus providing a useful combination of properties for biocatalysis. Family members share the common (α/β)_8_ TIM barrel fold with eight β-strands forming the barrel, surrounded by eight α-helices running antiparallel to the strands. The carboxy ends of the β-strands are connected to the α-helices by loops forming the active site. An additional N-terminal β-turn and two extra α-helices complete the structure (Fig. 1). Active-site residues occupy superimposable positions in AKR crystal structures and coenzyme binding is nearly identical. The substrate-binding cavity is mainly formed by residues that belong to the large and flexible loops 4 (α_4_-helix to β_4_-sheet), 7 (α_7_-helix to β_7_-sheet) and the C-terminus (Jez *et al.*, 1997) (Fig. 1A). Loop flexibilities provide the structural basis for the oftentimes relaxed substrate specificities. However, mobile loops also slow down the reaction rate (*k*_cat_ of max ∼30 s^−1^; e.g. Ma and Penning, 1999; Couture *et al.*, 2004; Kratzer *et al.*, 2006; Campbell *et al.*, 2013) and add complexity to structure–function relationships (Couture *et al.*, 2004), thereby impairing AKR application in biocatalysis. A previous study aimed at inverting the substrate specificities of two mammalian hydroxysteroid dehydrogenases that act on opposite ends of steroid hormone substrates. Swapping of all three large loops (4, 7, C) was necessary to achieve inversion. Point mutations of substrate-binding residues were not sufficient to reverse substrate specificities despite the close relatedness of the enzymes (67% sequence identity) (Ma and Penning, 1999). Recently, grafting of loops from human aldose reductase into a hyperthermostable alcohol dehydrogenase gave a hint that substrate and coenzyme binding by AKRs is not entirely independent (Campbell *et al.*, 2013). In the present study, we optimized an AKR for the reduction of bulky-bulky ketones by minimal loop engineering. The aim of the study was to accelerate the enzymatic reduction rate for bulky-bulky ketones without altering the high stereoselectivity of the wild-type enzyme. Large changes in enzyme activity prompted an analysis of kinetic data and (co)substrate binding. New results and findings from previously reported AKR crystal structures provided further insights into the connectivity of coenzyme and substrate binding.

**Fig. 1.**
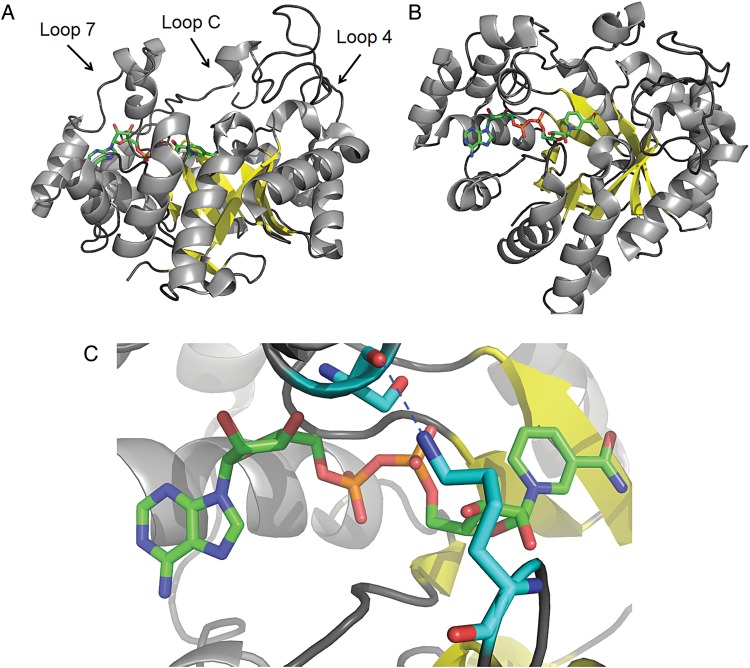
The overall fold of AKRs is shown using the example of *Ct*XR. The co-crystallized NAD^+^ is shown in green (PDB 1MI3). The three large loops are indicated by arrows (A). Structure rotated by 45 degrees around the x-axis (B). Close-up look of the “safety belt” formed by a hydrogen bond (dashed blue line) between Nξ of K24 to S223 (turquoise) (C). Visualized with PyMOL Molecular Graphics System, 0.99rc6, Schrödinger, LLC.

Loop 1 of *Candida tenuis* xylose reductase (*Ct*XR) has been previously shown to take part in substrate as well as coenzyme binding (Kavanagh *et al.*, 2002). We hybridized this loop with the homologous region of *Bos taurus* prostaglandin F synthase 2 (*Bt*PS2) (Suzuki *et al.*, 1999), a distinctively different AKR (sequence identity 39%; Blosum62). The native loop 1 of *Ct*XR is five amino acids in length (22–26) and carries a tryptophan at position 23. It was previously shown that aldehyde-preferring AKRs have exclusively tryptophans at positions homologous to 23 in *Ct*XR, whereas ketone-preferring AKRs harbor smaller amino acids at the respective position (Kratzer *et al.*, 2006). We replaced W23 and the subsequent lysine in *Ct*XR by the homologous *Bt*PS2 sequence. The alignment of *Bt*PS2 with *Ct*XR (Supplementary material) displays an insertion of three amino acids after position 23 in *Bt*PS2.

A multiple sequence alignment across AKR members suggests conservation of loop 1 with respect to length in enzymes with closely related substrate spectra (five amino acids in aldehyde and aldose reductases, mostly eight amino acids in hormone reductases and nine amino acids in steroid reductases acting on C–C double bonds (http://www.med.upenn.edu/akr/)). W23 and the subsequent K24 in *Ct*XR were replaced by the pentapeptide FAPRE found in *Bt*PS2. The redesigned *Ct*XR was expressed in *Escherichia coli* BL21 (DE3) and purified to apparent homogeneity by dye ligand chromatography (for details see the Supplementary material). The isolated enzyme was assayed in reductions of d-xylose, 2′-chloroacetophenone, 2′,4′-dichloroacetophenone and ethyl 4-cyanobenzoylformate and compared with the respective wild-type values (Table I). Substrates were chosen with regard to wild-type specificity and industrial relevance as previously reported (Kratzer and Nidetzky, 2007; Kratzer *et al.*, 2011). The natural substrate d-xylose was converted by the mutant enzyme with a *k*_cat_/*K*_m xylose_ value of 0.23 M^−1^ s^−1^, accounting for only 0.2% of the respective wild-type activity. The decreased catalytic efficiency (*k*_cat_/*K*_m xylose_) was mainly due to a 150-fold decreased turnover number and an ∼6-fold higher binding constant for xylose (*k*_cat_/*K*_m xylose_). Catalytic efficiencies for 2′-chloroacetophenone and 2′,4′-dichloroacetophenone reductions were similar for wild-type and mutant *Ct*XR. The bulky-bulky substrate ethyl 4-cyanobenzoylformate was, however, reduced with 64-fold higher catalytic efficiency by the mutant enzyme. The wild type's distinguished stereoselectivity in the reductions of 2′-chloroacetophenone and ethyl 4-cyanobenzoylformate (Kratzer and Nidetzky, 2007; Kratzer *et al.*, 2011) was retained in the mutant enzyme. Optical purities of (*S*)-1-(2′-chlorophenyl)-ethanol and ethyl (*R*)-4-cyano-mandelate were determined to be >99.9 and 99.8% ee (enantiomeric excess in percentage), respectively, by chiral high-performance liquid chromatography (Kratzer and Nidetzky, 2007; Kratzer *et al.*, 2011). Identical stereoselectivities of wild type and mutant suggest a similar orientation of substrate–NADH–enzyme complexes. Formation and breakup of ternary complexes in AKRs are thought to follow an ordered bi–bi mechanism, in which the coenzyme binds first and leaves last. The initial coenzyme binding triggers Loop 7 to fold like a ‘safety belt’ over the coenzyme and lock it by hydrogen bonds during catalysis (Jez *et al.*, 1997; Kavanagh *et al.*, 2002) (Fig. 1C). The association constant of coenzyme and enzyme is reflected by the specificity constant of the coenzyme (*k*_cat_/*K*_m NAD(P)H_). In a strictly ordered mechanism, where the coenzyme binds first and the carbonyl compound binds second, *k*_cat_/*K*_m NAD(P)H_ is independent of the second substrate (Segel, 1975). We determined a *k*_cat_/*K*_m NADH_ of 3.1·10^5^ M^−1^ s^−1^ for the reduction of xylose by *Ct*XR. Reductions of three aromatic ketones by wild-type *Ct*XR resulted, surprisingly, in 22–110-fold reduced *k*_cat_/*K*_m NADH_ values (Table I). The respective *k*_cat_/*K*_m NADH_ value for xylose reduction by the mutant *Ct*XR was 550 M^−1^ s^−1^ and increased 14–140-fold when aromatic ketones listed in Table I were used as second substrates. The variance in *k*_cat_/*K*_m NADH_ values strongly indicates a switch from ordered to random binding when either a substrate other than xylose is converted by the wild-type or the mutant enzyme is used as the catalyst. (The variance in *k*_cat_/*K*_m NADH_ spans two orders of magnitude across the tested substrates for the wild-type and mutant enzyme. Note that the variation in *k*_cat_/*K*_m NADH_ is hence significantly larger than a possible uncertainty resulting from incomplete substrate saturation.) The connectivity of NADH and carbonyl substrate binding was further investigated by saturation transfer difference (STD) ^1^H-NMR.

**Table I.** Apparent kinetic parameters of wild type and mutant for NADH-dependent reduction of a series of carbonyl substrates

Substrate (maximal concentration in mM)	Kinetic parameters^a,b,c^	Wild type	Mutant
Xylose (700)	*K*_m A_ (mM)	91^d^	540 ± 110
	*k*_cat app_ (s^−1^)	12^d^	0.08 ± 0.01
	*k*_cat_/*K*_m A_ (M^−1^ s^−1^)	132^d^	0.23 ± 0.02
	*K*_m NADH_ (µM)	39^d^	201 ± 35
	*k*_cat_/*K*_m NADH_ (M^−1^ s^−1^)	308 000^d^	550 ± 30
2′-Chloroacetophenone (10)	*K*_m A_ (mM)	13 ± 4	5 ± 2
	*k*_cat app_ (s^−1^)	0.8 ± 0.1	1.0 ± 0.2
	*k*_cat_/*K*_m A_ (M^−1^ s^−1^)	290 ± 30	370 ± 50
	*K*_m NADH_ (µM)	110 ± 25	70 ± 20
	*k*_cat_/*K*_m NADH_ (M^−1^ s^−1^)	8700 ± 400	7600 ± 500
2′,4′-Dichloroacetophenone (5)	*K*_m A_ (mM)	6 ± 2	4 ± 1
	*k*_cat app_ (s^−1^)	2.7 ± 0.3	2.0 ± 0.4
	*k*_cat_/*K*_m A_ (M^−1^ s^−1^)	840 ± 70	1010 ± 260
	*K*_m NADH_ (µM)	170 ± 50	80 ± 25
	*k*_cat_/*K*_m NADH_ (M^−1^ s^−1^)	14 300 ± 1300	8500 ± 900
Ethyl 4-cyanobenzoylformate (10)	*K*_m A_ (mM)	5 ± 2	4 ± 1
	*k*_cat app_ (s^−1^)	0.4 ± 0.06	17 ± 2
	*k*_cat_/*K*_m A_ (M^−1^ s^−1^)	100 ± 10	6400 ± 900
	*K*_m NADH_ (µM)	140 ± 25	150 ± 50
	*k*_cat_/*K*_m NADH_ (M^−1^ s^−1^)	2800 ± 100	77 000 ± 4000
Ethyl benzoylformate (10)	*k*_cat_/*K*_m A_ (M^−1^ s^−1^)	89 ± 5	6300 ± 800
Ethyl 2-chlorobenzoylformate (1.5)	*k*_cat_/*K*_m A_ (M^−1^ s^−1^)	350 ± 20	29 000 ± 4000
Ethyl 3-chlorobenzoylformate (1.5)	*k*_cat_/*K*_m A_ (M^−1^ s^−1^)	52 ± 4	3000 ± 80
Ethyl 4-chlorobenzoylformate (1.5)	*k*_cat_/*K*_m A_ (M^−1^ s^−1^)	1200 ± 100	56 000 ± 5000

^a^Uncertainties in *K*_m_ values are due to incomplete substrate saturation, i.e. low substrate solubilities or high absorption of NADH (>300 µM). *K*_m A_ is the *K*_m_ value of the carbonyl substrate.

^b^*k*_cat app_ were determined from initial rates at maximal substrate concentrations and 300 µM NADH.

^c^Catalytic efficiencies (*k*_cat_/*K*_m_) were calculated from the slope of the Michaelis–Menten plot where the rate is linearly dependent on the (co)substrate concentration and equals *k*_cat_[E]/*K*_m_.

^d^Data are taken from Kratzer *et al.* (2004). All data show mean values from three independent measurements.

In STD ^1^H-NMR, protons of the protein are saturated by spin diffusion after selective irradiation. Intermolecular saturation transfer leads to saturation of a bound ligand and is detected after dissociation from the enzyme. Signal intensities depend on the distance of the ligand protons to the protein surface, the residence time of the ligand in the binding pocket and the number of ligands that are magnetized during saturation time (Meyer and Peters, 2003; Fielding, 2007). Ligands that undergo reaction during the experiment provide STD effects from enzyme–substrate and enzyme–product complexes. Measurement of enzyme–substrate complexes requires conditions that allow the substrate to dissociate from the enzyme much faster than it reacts to products (Brecker *et al.*, 2006). In the present study, a lowered temperature of 10°C was used to maximize the signals of enzyme–substrate complexes and slow down catalysis (for further details see the Supplementary material). Figure 2 shows a global view of NADH–wild-type interactions in the binary complex (Fig. 2A; Supplementary Table SI) and ternary complexes with xylose, 2′-chloroacetophenone or 2′,4′-dichloroacetophenone (Fig. 2B–D; Supplementary Table SI in the Supplementary material). A comparison of STD effects obtained from the binary wild type–NADH complex and ternary complexes indicates that ribose- and nicotinamide protons are more tightly bound in the presence of xylose but less tightly bound in complexes with 2′-chloroacetophenone and 2′,4′-dichloroacetophenone (Fig. 2). The structural explanation for the observed STD effects is a loop that folds over the bound coenzyme in the carbohydrate region. Apo and holo crystal structures of *Ct*XR (PDB 1JEZ, 1K8C, 1MI3) show that loop 7 becomes ordered upon coenzyme binding and is held in place by hydrogen bonds between Nξ of K24–S223 (Fig. 1C) (Kavanagh *et al.*, 2002). Saturation transfer difference results suggest that the loop cannot fasten in the presence of bulky ketones. Furthermore, the closing anchor K24 is mutated in the redesigned *Ct*XR and observed STD effects on the bound NADH are hence lowest in ternary complexes of mutant enzyme, NADH and xylose (Fig. 2E; Supplementary Table SI) or 2′,4′-dichloroacetophenone (Fig. 2F; Supplementary Table SI.) Previous analysis of transient kinetic data for xylose reduction by wild-type *Ct*XR provided estimates of microscopic rate constants. The net rate constant of NAD^+^ dissociation turned out as rate-limiting *k*_cat_ (Nidetzky *et al.*, 2001). The ∼43-fold increased turnover number for the reduction of ethyl 4-cyanobenzoylformate by mutated *Ct*XR suggests a large change in microscopic rate constants as compared with the wild type-catalyzed reduction. The absence of a slow loop movement suggests rate enhancement by faster dissociation of NAD^+^ in the mutant. Furthermore, replacement of W23 in the wild type by the smaller phenylalanine and elongation of loop 1 provides more space for the accommodation of bulky-bulky ketones like ethyl benzoylformates. The production of optically pure mandelate esters is required in biocatalysis (Kratzer and Nidetzky, 2007). We hence probed the mutant *Ct*XR as biocatalyst for the reductions of ethyl benzoylformate, ethyl 2-chlorobenzoylformate, ethyl 3-chlorobenzoylformate and ethyl 4-chlorobenzoylformate. The obtained catalytic efficiencies were 43- to 83-fold higher when compared with the wild-type enzyme (Table I). The newly designed enzyme meets the process’ key requirements of absolute enantioselectivity and high turnover number (Kratzer and Nidetzky, 2007).

**Fig. 2.**
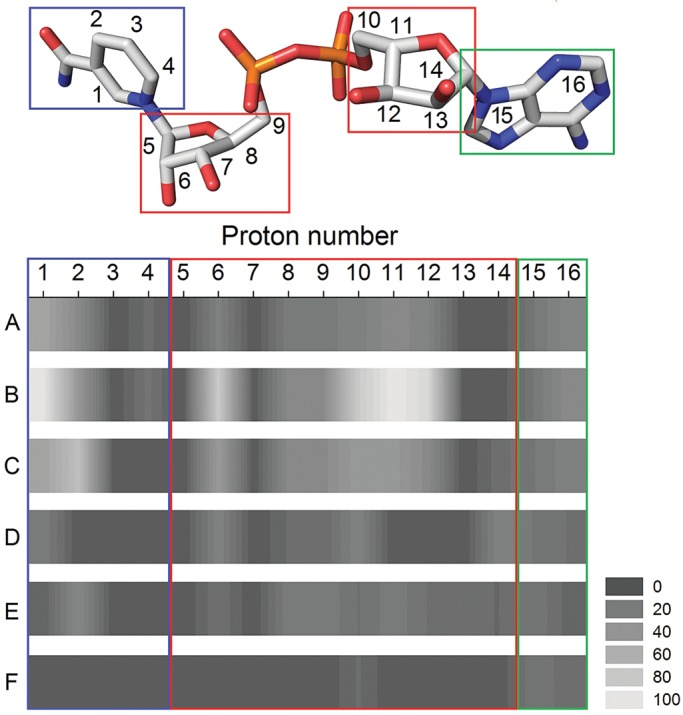
*Ct*XR-NADH interactions visualized by STD ^1^H-NMR. Wild type *Ct*XR•NADH binary complex (A) compared to wild type ternary complexes with xylose (B), 2′-chloroacetophenone (C), 2′,4′-dichloroacetophenone (D) and mutant ternary complexes with xylose (E) and 2′,4′-dichloroacetophenone (F). (STD effects are shown by shades of gray; tight binding effect of the respective proton is indicated by light gray (scored 100), no binding effect is indicated by dark gray (scored 0). Signals of nicotinamide protons 4-pro-*R* and 4-pro-*S* are summarized to signal 2. Due to uncertainties in precise proton assignment, effects are allocated to molecule domains. Data is shown in the Supplementary material).

## Supplementary data

Supplementary data are available at *PEDS* online.

## Funding

This work was supported by the Austrian Science Fund (FWF) [Elise Richter grant V191-B09 to R.K.; grant P-15208-MOB to B. N.]. Funding to pay the Open Access publication charges for this article was provided by the Austrian Science Fund (FWF).

## Supplementary Material

Supplementary Data
