# Supplementary material for: Acceleration of an aldo-keto reductase by minimal loop engineering
Source: Protein Eng Des Sel. 2014 Jul;27(7):245–8. doi: 10.1093/protein/gzu021 (PMC4064709; doi:10.1093/protein/gzu021)
Supplement: Supplementary Data [file supp_27_7_245__index.html]

Supplementary Data 

# Acceleration of an aldo-keto reductase by minimal loop engineering

## Supplementary Data

Supplementary Data

**Files in this Data Supplement:**

- Supplementary Data - Doc file
