# Supplementary material for: Acceleration of an aldo-keto reductase by minimal loop engineering
Source: Protein Eng Des Sel. 2014 Jul;27(7):245–8. doi: 10.1093/protein/gzu021 (PMC4064709; doi:10.1093/protein/gzu021)
Supplement: Supplementary Data [file supp_gzu021_gzu021supp.doc]

**Acceleration of an aldo-keto reductase by minimal loop engineering**

C. Krump^1^, M. Vogl^2^, L. Brecker^2^, B. Nidetzky^1,3^, R. Kratzer^1,3^

^1^Institute of Biotechnology and Biochemical Engineering, Graz University of Technology, Member of NAWI Graz, 8010 Graz, Austria

^2^Institute of Organic Chemistry, University of Vienna, 1090 Vienna, Austria

^3^To whom correspondence should be addressed. E-mail: [bernd.nidetzky@tugraz.at](mailto:bernd.nidetzky@tugraz.at); [regina.kratzer@tugraz.at](mailto:regina.kratzer@tugraz.at)

**Supporting Information**

*Site directed mutagenesis, enzyme production and purification*

*Methods of saturation transfer difference NMR Spectroscopy*

Fig. S1. Alignment of *Candida tenuis* xylose reductase (*Ct*XR) and *Bos taurus* prostaglandin F synthase 2 (*Bt*PS2).

Table SI. *Ct*XR-NADH interactions visualized as STD ^1^H-NMR intensities.

*Site directed mutagenesis, enzyme production and purification*

Site-directed mutagenesis and amino acid insertion were carried out by using inverse PCR as described elsewhere (Kratzer et al. 2006). The mutagenic oligonucleotide primers are listed below with the mismatched bases underlined.

Forward: 5´ TCGTGAACTCGCTAACGCTACTGCTGGTG 3´

Reverse: 5´ GGAGCGAAACA**T**CCGAAACCGATGGAAGGC 3´

To facilitate colony screening by restriction site analysis, a *Fok*I restriction site in the reverse primer was inserted by introducing a silent mutation (marked in bold). The authenticity of each mutagenised gene was confirmed by dideoxy sequencing. Recombinant wild type *Ct*XR was produced in *E. coli* and purified to apparent homogeneity using reported protocols (Mayr et al. 2000). Production of mutant *Ct*XR followed a slightly modified protocol. The expression of mutant *Ct*XR was induced by addition of 200µM isopropyl β-D-1-thiogalactopyranoside (wild type 125µM) with a concomitant reduction of cultivation temperature to 20°C (wild type 25°C). Purified mutant *Ct*XR migrated in SDS/PAGE as single protein band to exactly the same position as the wild type (data not shown).

B1 B2 ß1 loop 1 α1 ß2 loop 2 α2


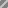

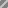


*Ct*XR 1 SASIPDIKLSSGHLMPSIGFGCW---KLANATAGEQVYQAIKAGYRLFDGAEDYGNEKEVGDGVKR 63

*Bt*PS2 1 DPKGQRVKLNDGHFIPVLGFGTFAPREVPKSEALEVTKFAIEAGFRHIDSAHLYQNEEQVGQAIRS 66

** ** * *** * * ** ** * * * * ** **

Mutated *Ct*XR 1 SASIPDIKLSSGHLMPSIGFGCFAPRELANATAGEQVYQAIKAGYRLFDGAEDYGNEKEVGDGVKR 66

Fig. S1. Alignment of *Ct*XR and *Bt*PS2 (matrix Blosum62, gap open penalty 12, gap extension penalty 4) with secondary structure. ß-Sheets are indicated as yellow arrows, α-helices as gray barrels. Mutated region in loop 1 is shown in red.

*Steady-state enzyme kinetics*

Unless mentioned otherwise, all experiments were performed at 25 °C in 50mM potassium phosphate buffer, pH 7.0. Initial rate measurements were carried out as described recently (Mayr et al. 2000), with 5% ethanol added as required to enhance the solubility of hydrophobic carbonyl compounds. Kinetic parameters for enzymatic xylose reduction by NADH were not affected by the added ethanol. The initial rates were recorded immediately after preparation of the α-keto esters to avoid their non-enzymatic decomposition in aqueous solution (Kratzer and Nidetzky, 2007). Unless indicated, they were obtained under conditions in which [one substrate] was varied and the [other substrate] was constant. The enzyme concentration in the assays was in the range of 0.5 to 100µM, depending on the activity towards the respective substrate. Appropriate controls containing enzyme and co-enzyme, or the substrate and co-enzyme were determined under conditions otherwise exactly identical to the enzymatic assay. If required, the initial rates were corrected for blank readings. Kinetic parameters were obtained from unweighted non-linear least-square fits of eq. 1 to the experimental data using the program SigmaPlot 2004 (for Windows, version 9.0). In eq 1,

v = *k*_cat_[E][A]/(*K*_m_ + [A]) (1)

v is the initial rate, [E] is the molar concentration of the enzyme subunit (wild type 36.0kDa; mutant 36.3kDa), [A] is the substrate or coenzyme concentration, *k*_cat_ is the turnover number (s^-1^) and *K*_m_ is an apparent Michaelis-Menten constant. The obtained uncertainties in *K*_m_ values are due to incomplete substrate saturation i.e. low substrate solubilities or high absorption of NADH (>300µM). Corresponding *k*_cat_ values were determined from initial rates at maximal substrate concentrations and 300µM NADH (*k*_cat app_). Catalytic efficiencies (*k*_cat_/*K*_m_) were calculated from the slope of the Michaelis-Menten plot where the rate is linearly dependent on the (co)substrate concentration and equals *k*_cat_[E]/*K*_m_ ([(co)substrate] « *K*_m_). Data show mean values from three independent measurements.

*Methods of Saturation Transfer Difference (STD) NMR Spectroscopy*

Samples were prepared in 0.7mL potassium phosphate buffer (50mM, D_2_O) at a pD-value of 7.0. Substrate (xylose, 2'-dichloroacetophenone or 2',4'-dichloroacetophenone) and NADH were used in concentrations of 2.5mM. The acetophenones were pre-dissolved in CD_3_OD (end concentration 5%) due to low solubilities in buffer. Reactions were started by the addition of enzyme. Concentrations of wild type and mutant *Ct*XR in reactions with xylose or 2'-chloroacetophenone were 1 and 0.4µM, respectively. Enzyme concentrations were halved when 2',4'-dichloroacetophenone was used. All ^1^H and STD NMR spectra were measured on a Bruker (Rheinstetten, Germany) DRX-600 AVANCE spectrometer at 600.13MHz using triple resonance 5mm inverse probe. STD spectra were recorded as described earlier (Brecker et al. 2006). On resonance measurements were performed at -2.0ppm and off resonance measurements at 40.0ppm. Saturation time of the *Ct*XR enzymes was 2.0s and the number of scans for the experiments varied between 128 and 256 at a temperature of 10°C. Short measurement times were used to account for the reaction progress with changing concentrations and to allow STD NMR during the transformations. The signal of one proton in comparable experiments was set to 100 (here proton number 15, Figure 2, Table SI) and relative intensities were determined (Brecker et al. 2006; Brecker et al. 2008). For the measurement of STD effects on NADH in ternary *Ct*XR·NADH·substrate between 16 and 64 proton measurements were performed in regular intervals over a total time of 3-8h. Each spectrum was recorded with 64 scans. Reported STD signals were recorded under conditions of ≤5% product formation.

Table SI. *Ct*XR-NADH interactions visualized as STD ^1^H-NMR intensities. Wild type *Ct*XR•NADH binary complex (A) compared to wild type ternary complexes with xylose (B), 2'-chloroacetophenone (C), 2',4'-dichloroacetophenone (D) and mutant ternary complexes with xylose (E) and 2',4'-dichloroacetophenone (F). Proton 15 was used as reference point in each experiment. The signal of proton 15 was set to 100 and relative intensities of the other protons were determined within one experiment. Signals of nicotinamide protons 4-pro-*R* and 4-pro-*S* are summarized to signal 2.

| **Proton No** | **1** | **2** | **3** | **4** | **5** | **6** | **7** | **8** | **9** | **10** | **11** | **12** | **13** | **14** | **15** | **16** |
| --- | --- | --- | --- | --- | --- | --- | --- | --- | --- | --- | --- | --- | --- | --- | --- | --- |
| **A** | 330 | 190 | 0 | 60 | 0 | 140 | 0 | 120 | 120 | 140 | 200 | 155 | 0 | 0 | 100 | 170 |
| **B** | 630 | 260 | 0 | 85 | 0 | 505 | 0 | 190 | 190 | 505 | 640 | 570 | 0 | 0 | 100 | 200 |
| **C** | 310 | 450 | 0 | 0 | 0 | 275 | 0 | 195 | 195 | 275 | 270 | 190 | 0 | 65 | 100 | 145 |
| **D** | 125 | 0 | 0 | 0 | 0 | 150 | 0 | 55 | 55 | 150 | 0 | 25 | 0 | 150 | 100 | 75 |
| **E** | 30 | 180 | 0 | 0 | 0 | 75 | 0 | 125 | 125 | 75 | 120 | 75 | 60 | 50 | 100 | 40 |
| **F** | 0 | 25 | 0 | 0 | 0 | 0 | 0 | 0 | 0 | 55 | 0 | 0 | 0 | 0 | 100 | 60 |

Brecker, L., Schwarz, A., Gödl, C., Kratzer, R., Tyl, C.E. and Nidetzky, B. (2008) *Carbohydr. Res.*, 343, 2153-2161.

Brecker, L., Straganz, G.D., Tyl, C.E., Steiner, W. and Nidetzky, B. (2006) *J. Mol. Catal. B: Enzym.*, 42, 85-89.

Kratzer, R., Leitgeb, S., Wilson, D.K. and Nidetzky, B. (2006) *Biochem*. *J*., 393, 51-58.

Kratzer, R. and Nidetzky, B. (2007) *Chem. Commun.*, 10, 1047-1049.

Mayr, P., Brüggler, K., Kulbe, K.D. and Nidetzky, B. (2000) *J. Chromatogr. B*., 737, 195-202.
